# Exploring willingness of elder Chinese in Houston to participate in clinical research

**DOI:** 10.1016/j.conctc.2016.06.006

**Published:** 2016-06-24

**Authors:** Logan R. Thornton, Rossybelle P. Amorrortu, Daniel W. Smith, Arch G. Mainous, Sally W. Vernon, Barbara C. Tilley

**Affiliations:** a The University of Texas Health Science Center at Houston (UT Health) School of Public Health, Department of Health Promotion and Behavioral Sciences, 1200 Hermann Pressler, Houston, TX, 77030, USA; b The University of Texas Health Science Center at Houston (UT Health) School of Public Health, Department of Biostatistics, 1200 Hermann Pressler, Houston, TX, 77030, USA; c Medical University of South Carolina, Department of Psychiatry and Behavioral Sciences, 100 Doughty Street, Charleston, SC, 29425, USA; d University of Florida, Department of Health Services Research, Management and Policy, Health Science Center, PO Box 100195, Gainesville, FL, 32610, USA

**Keywords:** Minority, Elder adults, Trial participation, Trust, Chinese, Recruitment

## Abstract

**Background and objectives:**

Inadequate minority participation in clinical research can threaten the applicability and strength of scientific findings. Previous research suggests that trial participation rates are lowest among Asian Americans, compared to other groups. This study explored barriers to clinical research participation among elder Chinese living in Houston, Texas. Additionally we administered the Trust in Medical Researchers Scale (TIMRS), used previously in researching trust in medical researchers as related to research participation.

**Design:**

In this mixed methods study, a semi-structured interview, including the TIMRS were administered to 30 adults of Chinese ancestry aged 50 years or older recruited from a Chinese community center. Interviews were conducted in English, Mandarin and Cantonese and independently coded and analyzed using thematic content analysis. TIMRS scores were calculated for participants.

**Results:**

Participants were 70% female, 70% were 60 or elder, all were foreign born and on average lived in the US for 21.8 years. Participants perceived risks to research participation and preferred language concordant research staff. Interviewees were more willing to participate if they perceived personal and community health-related benefits. The overall TIMRS score was 23.9 (±5.0), lower than the overall TIMRS for Whites in a previous study (P < 0.001).

**Conclusions:**

The barriers and facilitators to research participation confirmed previous research among Asians. Our participant TIMRS scores were consistent with decreased levels of trust observed in the original TIMRS study for African Americans as compared and lower than Whites. Employing strategies that utilize language concordant staff who build trust with participants may aid in recruiting elder Chinese, especially if the research is personally relevant to those being recruited.

## 1 Introduction

Underrepresentation of racially and ethnically diverse groups in medical research is an ongoing problem [1], [2], [3], [4]. Participant diversity in medical research allows researchers to generalize their findings more broadly and to analyze responses to experimental interventions received by different racial and ethnic groups [5], [6], [7], [8], [9]. When participants lack diversity, researchers are limited in their ability to draw scientifically rigorous conclusions about the efficacy of experimental interventions. The U.S. Census Bureau projects that the proportion of the population over age 65 will continue to increase and the population in general will continue to increase in diversity [10]. With Medicare bearing the health care costs for most of those over age 65, interventions to maintain or improve the health of this population are increasingly important. The proportion of elder Asians in the US is also expected to increase [11].

Asian Americans are disproportionally affected by several debilitating diseases including chronic hepatitis B, HIV/AIDS, and tuberculosis [12]. Currently, Asian Americans experience higher rates of morbidity and mortality from liver and stomach cancer when compared to non-Hispanic whites [13]. Despite the disease burden, clinical trial participation rates among Asians were reported to be lowest compared to other racial and ethnic minorities [14], [15].

In general, medical researchers often confront challenges when recruiting minority participants, especially elder adults [16]. Numerous studies have reported barriers to enrollment including lack of participant trust [17], [18], [19], [20], ineligibility due to comorbid conditions [21], [22], and lack of information about clinical study opportunities [23]. We reviewed the extant research examining barriers and facilitators for minority participation. The majority of studies have focused on barriers among African Americans relative to other racial and ethnic groups [3], [4], [24], [25].

The literature gives some insight as to why there is low participation and suggests that Asian have similar barriers to trial participation as other ethnic groups such as fear of side effects from experimental treatments [26], limited knowledge about clinical research [27], [28], [29], language barriers [27], [30], [31], and mistrust [28]. A 2005 study reported that, compared to non-Asians, elder Asian immigrants were more influenced to participate in research by children, landlords, physicians and the media [32]. Personal gain [31], involving the patient’s family in the decision making process [27], and receiving a recommendation to participate by trusted relatives or family physicians [27] have all been identified as possible motivators for research participation among Asians. Few studies take the Asian population’s heterogeneity in to account [33] or characterize facilitators and barriers exclusively for the Chinese population, the largest Asian subgroup in the U.S. [28], [29].

We explored the patient perspective on willingness to participate in medical research among a sub-group of elder Chinese living in Houston, TX. We addressed the patient perspective via in-depth interviews supplemented by a psychometrically validated self-report scale. The present study is the first to administer the Trust in Medical Researchers Scale (TIMRS) in this population. This validated scale has examined mistrust in medical researchers in African American and White community residents [20]. The scale measures the impact of researcher honesty and participant deception, two key domains in researchers mistrust. Since much of the body of evidence exploring mistrust in minority populations is overwhelmingly qualitative in nature we wanted to supplement our qualitative study with a scale to quantify trust in medical researchers in the elder Chinese population.

## 2 Methods

### 2.1 Design

This study applied a mixed methods approach (in-depth interviews and a quantitative scale). A qualitative research approach was selected as a useful framework for studying patients’ beliefs and willingness to participate in medical research [34]. Qualitative research explores research participation from the participant perspective and generates theories to be tested further in more traditional study designs. Qualitative research cannot determine causal relationships [35]. The qualitative analyses of the in-depth interviews were supplemented with quantitative data from the TIMRS.

### 2.2 Participant recruitment

Participant recruitment was facilitated by an established relationship between the University of Texas Health Science Center at Houston (UTHealth) and the Houston (TX) Chinese Community Center, which provides a variety of services ranging from childcare to senior services. Community center staff were familiar with regular visitors to the center so they recruited a purposive sample of 30 participants based on the study eligibility criteria, i.e., of Chinese ancestry and aged 50 years or older. Sample size was determined based on qualitative research employing semi-structured in-depth interviews to achieve saturation of themes [36] and develop theories for future testing. The participants were specifically recruited from programs oriented towards the elder population such as the Adult Day Program and the Senior Companionship Program. This age group was selected to gain more information about this Asian group’s willingness to participate in research evaluating treatments.

### 2.3 Study instruments

The research project received approval through the UT Health Committee for Protection of Human Subjects (HSC-SPH-11-0608). The interview guide was developed in English based on previous research that studied knowledge and awareness about the goals of medical research; trust of health care providers and the health care system; and attitudes toward volunteering in research [20], [37], [38], [39]. The interview guide included three open-ended questions about the participant’s knowledge and awareness of medical research. The remaining five questions asked the participant to rate on a 10-point scale their willingness to participate in a hypothetical clinical trial within the next three months where 10 represented very likely and 0 represented not at all likely. Specifically, we asked whether compensation, free transportation, flexible clinic hours, Asian study staff, native language, or translation services increased their willingness to participate. We also administered the TIMRS to participants [20] a validated scale measuring trust in medical researchers. Items on TIMRS can be separated into two subscales: participant deception (items 1–6) and researcher honesty (items 7–12). Participant deception items relate to the participant’s beliefs about being deceived or misled by medical researchers. The researcher honesty subscale relates to participant’s beliefs about the honesty of the researchers in explaining aspects of clinical trial participation. The TIMRS scores and subscale scores range from 0 to 48 with higher scores indicating a greater level of trust in medical researchers. A Chinese graduate student translated the interview guide and the TIMRS into Chinese. A different Chinese student, unfamiliar with the project, back translated the instruments into English. We did not find discrepancies between the forward and back translations. The interview guide and TIMRS also underwent brief cognitive pretesting prior to use and no problems were noted [40].

### 2.4 Procedures

The Chinese graduate student that translated the interview guide also conducted interviews in Mandarin. A second Chinese graduate student was hired to conduct interviews in Cantonese. Two non-Chinese students and a non-Chinese study coordinator conducted the English interviews. All interviewers were trained in qualitative interview methods by an experienced qualitative researcher. Participants were read a consent form and provided verbal consent prior to the beginning of the study. The 20–30-min, in-person interviews were conducted in a private room at the community center.

The TIMRS was administered after the qualitative questions. Participants were read each question aloud and asked to point to a card indicating their response. The scale items were answered on a 5-point Likert-type scale with answer choices ranging from 1 = strongly disagree to 5 = strongly agree. The participants were given the option to interview in Mandarin, Cantonese or English. Seventeen interviews were conducted in Mandarin, 11 in English and two in Cantonese. All interviews were digitally recorded, and notes were made. Participants were compensated with a $20 Walmart gift card.

### 2.5 Data analysis

Interviews were professionally translated (if non-English) and transcribed by an outside company. All transcripts were independently coded by the first and second authors using thematic content analysis. Themes were not developed *a priori*. Codes were categorized into sub-themes, identified in the interview questions, along with definitions and supporting quotes. Both investigators developed separate codebooks and, through discussion, reached consensus on themes that best illustrated the ideas communicated in the interviews. We used ATLAS.ti software version 7 for coding and the Consolidated Criteria for Reporting Qualitative Research (COREQ) to present the qualitative results [41], [42]. The TIMRS data were analyzed using SAS software version 9.3 [43]. Three scale scores were calculated from the TIMRS including overall trust in medical researchers, participant deception and researcher honesty. The details on how to calculate scale scores are reported elsewhere [20].

We compared the mean overall trust scores and trust score subscales for our elder Chinese participants to those reported by White and African American participants in the original TIMRS scale development study [20] using separate one-way analyses of variance (ANOVAs). In the presence of a significant one-way ANOVA (giving us weak protection of alpha) we planned two, 2-sample t-tests to compare the mean trust scores of our participants separately to scores from White and African Americans as reported in the Mainous study [20]. We used Bonferroni adjusted p-values of 0.025 to adjust for the two comparisons within the overall trust scale score or sub-scale scores. We also ran a logistic regression model to examine the association between our participants’ overall trust score and their dichotomized likelihood of participation in future medical research (0–3, 4–10) adjusting for any covariates significant at the 0.2 level [44]. We chose 0.2 to adjust for any variables weakly associated with likelihood of participation. Years in the United States was collected as a continuous variable. Other covariates were collected as binary (gender) or as categories. Because our sample size was small and some categories were sparse we dichotomized the two categorical covariates, age group [50–59, 60+] and education [high school or less, some college or more].

## 3 Results

A total of 30 Chinese participants born outside of the U.S. were interviewed, of whom 21 (70%) were female. The majority of participants (70%) were 60 years or older and on average reported living in the United States for 22 years (s.d. 13.29). Almost half of the participants (46%) held a college or advanced degree, 23% had some college education, 20% completed high school, and only 10% reported some high school or less. The majority of participants reflected a general but limited understanding of medical research and clinical trials. When asked how they would define medical research to a friend, most participants acknowledged that medical research was a method to advance medicine, to help people, and to develop treatments. When asked about past trial participation, three respondents reported experience with medical research projects. When probed about their research experience, only one participant described an actual research protocol.

### 3.1 In-depth qualitative interviews

Three themes emerged from the in-depth qualitative interviews; negative perceptions of research as inherently risky; importance of language concordance between study staff and participants; and benefits gained from participation. Quotes from the participants are used to illustrate the themes.

In general, approximately two-thirds of our participants expressed concerns about participating in medical research. Their primary concern appeared to be avoidance of risk. Concerns about safety, side effects, the uncertainty of experimental treatments, and invasive study procedures were expressed during the interviews. Our participants defined risk as doing harm and particular concerns were identified about studies that used radiation, x-rays, surgical technologies, or experimental drugs. Participants were explicit about the kinds of risks and the resulting side effects.

*“The first thing to consider is safety reasons. I feel that it’s important that being a subject doesn’t cause side effects or whatever. I think I need to be guaranteed and there needs to be a little bit of confirmed data. That is, at our age, the first thing is, of course, health. So I think we need to first guarantee that there is no harm participating in this.”*

Additionally, participants viewed procedures that introduced a foreign substance or object to be invasive. Pharmaceutical drugs were also described as foreign substances and presumed to be more dangerous than natural ones. One participant said

*“Deciding factor is anything that do with man-made drugs, that I will not participate. If it is about nutrition or nutrients, that I will think about it. But not man-made drugs, I don’t believe in man-made drugs.”*

Some participants seemed to base their fears of participation on reports from others, or beliefs they had acquired about the relative dangers of medical procedures.

*“I got to see whether that thing is harmful. Because we don’t know exactly how medical [medicine] work, but we have heard too many things, like x-ray is harmful to you, if you take too many mammograms in one year, it will be harmful. So you will think about it if you don’t know the harms.”*

Some participants would only consider enrolling if their illness could not be ameliorated with a medication that has been approved for public use for a period of time or a treatment for which tangible results are visible. Perceived risks operated as a hypothetical barrier to enrolling in research studies. Participants perceived invasive studies, non-natural therapeutic agents, and possible side effects as risky and as a result, made them less likely to participate in medical research.

Respondents consistently reported that speaking the same language as the research staff was important. Willingness to participate in studies was clearly facilitated by language concordance. While few participants specifically stated that they preferred to speak Chinese, the majority noted that staff who spoke their language would make participation more likely in other studies. Speaking the same language made respondents feel comfortable, because, for many, English was their second language. Participants believed that fewer misinterpretations occurred when the same language was spoken and, in some cases, when there was ethnic concordance.

*“I can communicate with Asian health care workers, I can explain my conditions in more details and I can hear what the doctors say more clearly as well.”*

*“That will be a plus. See people like me, English is not [good]. Especially the medication’s name, illness name, diseases, very difficult to understand. If there is Asian people over there, they translate it right away, it saves a lot of trouble.”*

Interestingly, for some participants, as long as study staff spoke the same language (either Mandarin or Cantonese) their race/ethnicity did not matter. When probed further about other factors that would facilitate enrollment, respondents named clear communication as more important than staff demographics. For some respondents, translators facilitated participation for the same reasons. Participants felt that translators helped them understand often-difficult medical terminology, although a few thought it too cumbersome to arrange for translators during an office visit. Others were concerned that translators may not reliably express what the participant was trying to convey.

Participants expressed a greater willingness to enroll in studies if they perceived personal or community health-related benefits from participation. Many reported that research must address the participant’s health status, the well-being of family/loved ones, or the broader community in order for them to strongly consider participation. This quote illustrates how willing they are

*“Willingness depends on what disease I have. For example let’s say you research a particular aspect and if I know this is a disease that I have, I will go.”*

A few participants reported altruistic motivations for considering trial enrollment. They commented that even if the study was not personally relevant, it would advance the body of science so that cures and treatments could be made available in the future for all people. Among those endorsing altruistic motivations for participating in research, most respondents had positive expectations for the knowledge gained from trials. The need for personal gain was not apparent; indeed, benefits for unknown others were also seen as a benefit of participation.

“If I take the trial, and maybe later on it will benefit a lot of people. It doesn’t matter what happens to me, but I want to let people have the good result.”

### 3.2 Trust in Medical Researchers Scale findings

The quantitative portion of the study examined the level of trust in medical researchers among the elder Chinese participants living in Houston. The overall TIMRS score and sub-scale scores for our participants are reported in Table 1. Table 1 also gives the TIMRS scores from the original study [20].

**Table 1:** Comparison of the overall Trust in Medical Research Scale (TIMRS) and sub-scale scores between the current study of Asians and the previous study of African Americans and Whites*.

	Current Study	Mainous Study		Comparison (Current Study vs. Mainous Study)	
Trust Scores	Chinese	African American	White	Chinese vs. African American**	Chinese vs. White**
Mean ± SD	(N = 30)	(N = 105)	(N = 319)	P-Value	P-Value
Overall Trust	23.9 ± 5.0	24.1 ± 6.9	28.7 ± 5.6	0.86	<0.001
Researcher Honesty	26.7 ± 7.3	26.3 ± 7.7	29.7 ± 6.1	0.80	0.01
Participant Deception	21.1 ± 7.5	22.0 ± 7.6	27.8 ± 6.3	0.57	<0.001

* All P-values obtained from ANOVAs were <0.001.

** P-values were obtained from 2-sample *t*-test between Chinese and White or African American.

When participants were asked about their likelihood of participation in medical research within the next 3 months, 70% of the participants responded that they had some likelihood of participation. We also examined the association between the overall TIMRS score and likelihood of future participation using a logistic regression model. We found that after adjusting for age and number of years living in the United States, participants with higher overall trust scores were more likely to participate in a research trial within the next three months; (OR = 1.58, 95%CI 1.01–2.48). See Table 2.

**Table 2:** Association of overall trust in medical researchers and the likelihood of participation in a clinical trial within the next three months.

Covariates	Beta	SD	P-value	Adjusted OR	95% CL
Overall trust score	0.46	0.23	0.05	1.58	1.01–2.48
Age (60 + vs. 50–59)	−2.44	1.26	0.05	0.01	<0.001–1.05
Number of years living in the US	0.06	0.05	0.20	1.06	0.98–1.16

Note: The outcome variable (willingness to participate) was modeled as likely to participate in a future clinical trial within the next three months (Y = 1).

## 4 Discussion

Our study used a mixed methods approach to measure trust in medical research and researchers among an elder purposive sample of Chinese Americans living in Houston and to examine their willingness to participate in clinical trials. Although our study confirmed previous findings from Chinese groups [28] regarding the importance of language concordance and personal benefits this study extended those findings by emphasizing the importance of trust in research participation. Language issues have been identified as a recurrent barrier to research participation by Chinese and Latino samples [27], [39], [45]. When probing our participants about facilitators to research participation, they clearly expressed a preference for language-concordant interactions with potential research personnel. Translators were seen as a less desirable alternative than research staff who spoke their language. The issue of racial concordance among our participants seemed considerably less important than language concordance, which has been an issue in previous samples using different minority populations [46], [47].

Among our participants, motivation to participate appeared to be closely linked to participants’ perceptions that the research activity would provide a benefit to them personally, to their families, or to the larger overall Chinese community. This is consistent with some previous research on Chinese and African Americans [28], [48]. However, when asked about facilitators to research participation our participants did not appear to mention free screening or financial compensation, as has been seen in Latino samples and other Asian groups [24].

In line with previous research findings [39], [48], [49], our participants expressed perceived risks including concerns about the efficacy and safety of treatments, as barriers to participation. The findings of the importance of trust dovetail with the construct of perceived risks and bring to the fore the importance both communication and trust between the potential participant and the medical researchers. African Americans tended to cite the Tuskegee syphilis study, where non-minority researchers intentionally withheld treatment for syphilis from African American male participants, as the basis for their mistrust [49]. Latinos reported fears of inadequate safeguards and underqualified physicians (perhaps based on their experiences in their native countries) [39], [50]. Participants in our study believed the types of procedures (invasive/x-ray related interventions) and pharmaceutical (as opposed to “natural”) chemical agents conferred excessive risk for side effects. Participants trusted agents, mainly peers or family, to provide accurate information about medical research. Researchers, however, need to persuade participants that they were not going to be harmed in the name of science. Without such reassurance, our participants were less willing to engage in research.

Finally, although mistrust of research and researchers was not explicitly stated by the respondents, the comments about risk and corresponding protection of subjects implicitly suggested it. This concern was reinforced by the low TIMRS overall scores on trust in medical researchers. Interestingly, the trust scale scores reported by our participants are similar to the overall trust score of 21.1 reported by African Americans in a study by Mainous et al. [20] and lower than the Whites in that study. The study by Diaz et al. [51] reported an overall trust score of 26.5 among African American college students which was slightly higher than the trust scores reported by our participants. These findings point to similar levels of trust in medical researchers by these two minority groups. The low trust scores found may help explain the concerns expressed by participants regarding research risks. Participants were more willing to participate in studies testing efficacious drugs, such as antibiotics, but less willing to consider studies investigating new therapeutic agents. As was expected when examining the association between trust in researchers and the likelihood of future participation, participants with higher levels of trust were more likely to state that they would participate in a research study within the next three months. Additionally, while a high proportion of our study participants said they would participate in a clinical trial if asked, which is consistent with other studies [52], [53], this is likely an overestimation of actual study participation.

### 4.1 Limitations

As with all studies, our interpretations of the data are constrained by several factors. We had limited involvement in research subject selection, which could have resulted in selection bias by the community center staff. We had no knowledge of the current health status of the participants and did not elicit this information. It is likely that our participants were recruited from a relatively health population since they were able to attend activities at the Houston Chinese Community Center. Our study questions could have been more salient to those who were ill, which may have influenced the responses about potential participation in clinical trials. Because our participants were elder and all lived in Houston, we cannot generalize to younger Chinese adults or to elder adults living in other geographic areas. In addition, the use of inexperienced, although trained, graduate students as interviewers may have affected the quality and consistency of the interviews; however, the interview guide was designed to minimize these factors. It is also possible that the standardized order of the questions might have introduced some social desirability bias by priming participant responses.

## 5 Conclusions

Asians are under-represented in research studies [15], [54], [55], [56], [57], as are most minority groups [14], [58], [59]. Improved understanding of motives to participate in research across minority groups may aid researchers in tailoring recruitment plans to help redress this under-representation. Currently, RECRUIT (Randomized Recruitment Intervention Trial), a recruitment intervention to increase minority participation in clinical trials, is being tested in four randomized clinical trials across the United States. Study findings were used to inform the patient enrollment strategies used in RECRUIT. Specifically, the consistency of findings on trust in medical researchers reinforced our finding from studies of other minorities [20] and the importance of building trusting relationships with potential minority participants. Building a solid base of information about motivators and barriers to research participation by an under-represented minority group could further inform medical study design and recruitment strategies. Given the limited evidence-based approaches to enrolling Chinese participants, we must rely on the authentic voices that describe what would encourage study participation. In this way, researchers can be more economical and targeted with their resources to engage this population and use this information to develop more rigorous trials of approaches to encourage minority enrollment.
